# Defunctioning Ileostomy After Low Anterior Resection of Rectum: Morbidity Related to Fashioning and Closure

**DOI:** 10.3390/medicina60111864

**Published:** 2024-11-14

**Authors:** Nikolaos Gouvas, Dimitrios Manatakis, Christos Agalianos, Nikoletta Dimitriou, Ioannis Baloyiannis, George Tzovaras, Evangelos Xynos

**Affiliations:** 1 Department of General Surgery, Medical School, University of Cyprus, Nicosia 2404, Cyprus; dimitriou.nikoletta@ucy.ac.cy; 2 Department of General Surgery, Naval & Veterans Hospital, 11521 Athens, Greece; dmanatak@yahoo.gr; 3 Department of General Surgery, Naval & Veterans Hospital, 73200 Chania, Greece; xagali@gmail.com; 4 Department of General Surgery, University Hospital of Larissa, 41334 Larisa, Greece; balioan@hotmail.com (I.B.); gtzovaras@hotmail.com (G.T.); 5 Department of General Surgery, Creta Interclinic Hospital, 71304 Heraklion, Greece; exynos@gmail.com

**Keywords:** defunctioning ileostomy, rectal cancer, morbidity, ileostomy reversal

## Abstract

*Background and Objectives*: The aim of this study was to assess any predisposing factors to the morbidity of fashioning and reversal of diverting ileostomy in a prospective cohort of patients who have undergone TME and low colo-rectal or colo-anal anastomosis for rectal cancer. *Materials and Methods:* Consecutive patients with rectal cancer undergoing low anterior resection and a defunctioning loop ileostomy in three surgical units from 2016 to 2020 were included in the study and retrospectively analyzed. *Results*: One hundred eighty-two patients from three centres were included. Ileostomy-related mortality was 0.5%, attributed to renal failure.. Ileostomy-related morbidity was 46%. Postoperative ileus was seen in 37.4%, and dehydration in 18.8% of the patients. The readmission rate for ileostomy-related reasons was 15.4%. Stoma care was problematic in 15.7% or poor in 7% of the cases. Advanced age, male gender and obesity were independent risk factors for ileostomy-related morbidity. Ileostomy was reversed in 165 patients. The morbidity in 165 patients was 16%. Ileus was seen in 10.3%, anastomotic leak in 4.8% and wound infection in 12.7% of the cases. One patient died because of an anastomotic leak. No predisposing factors that affect the outcomes of ileostomy closure were identified. *Conclusions*: Diverting ileostomy-related morbidity is high. Life threatening dehydration and renal failure from ileus is more commonly seen in elderly, male and obese patients and should be anticipated. Ileostomy closure-related morbidity is low.

## 1. Introduction

The low anterior resection of the rectum (LARR) with total mesorectal excision (TME) is the procedure of choice for rectal cancer with preservation of anal sphincters. Anastomotic leakage is a severe complication reported in rates even higher than 20%, and a cause of mortality [1,2,3,4,5,6,7,8]. Anastomotic leak is seen in similar rates after the open or laparoscopic approach [9,10,11]. In addition to the location of the anastomosis, several other factors predispose for anastomotic leak, such as the male gender, history of chronic cardiovascular or respiratory disease, obesity and neo-adjuvant chemoradiotherapy [3,4,5,12,13,14]. Anastomotic leak is associated with increased immediate postoperative morbidity, in terms of pelvic sepsis and need for re-intervention, impaired long-term oncological outcomes and poor defaecatory function and quality of life [3,6,15,16,17].

There is substantial evidence that the addition of a defunctioning stoma to LARR reduces the rate and severity of anastomotic leak, as well as the rate of reoperation, or even mortality [18,19,20,21]. Diverting either ileostomy or colostomy is equally effective in preventing anastomotic leak [19,20,21,22]. However, morbidity related to the stoma formation is high [18,19,23,24,25], as is morbidity after stoma reversal [21,26,27]. In particular, ileostomy is associated with dehydration, ileus, readmissions and dermatitis, whilst anastomotic leak and reoperations after ileostomy closure are accounted in approximately 4% and 8%, respectively [25,27]. An additional factor of morbidity is a temporary defunctioning stoma, to become permanent because of complications, mostly anastomotic, from the initial LARR, thus preventing reversal [28].

There are clear benefits of fecal diversion in some, but not all, patients with LARR. Because of ileostomy-related morbidity, it is useful to thoroughly study the complications that are associated with diversion ileostomy, as well as to prevent them by reserving defunctioning ileostomy for those patients at high risk to develop anastomotic leak after LARR. Alternatively, alternative types of diversion, namely colostomy, can be offered to those prone to develop ileostomy-related severe complications. The present retrospective study aimed to further elucidate the issue of complications after both the fashioning and closure of defunctioning ileostomy in patients undergoing TME surgery for low rectal cancer and identify factors predisposing to ileostomy-related overall morbidity.

## 2. Materials and Methods

Consecutive patients with rectal cancer, with its lower border located at 10 cm from the anal verge, undergoing LARR and a defunctioning loop ileostomy in four surgical units (Naval & Veterans Hospital of Athens, Creta Interclinic Hospital, University Hospital of Larisa, Nicosia General Hospital) from 2016 to 2020 were included in this study and retrospectively analyzed. Patients with colonic resection for anything other than low rectal cancer pathology and loop ileostomy were excluded, as were those with a diverting colostomy.

TME was attempted in all patients either with the open or laparoscopic approach. Colo-rectal or colo-anal anastomosis was fashioned with the use of a circular stapling device or transanally by manual suturing. The decision for the type and the necessity of a defunctioning stoma was made by the operating surgeon preoperatively in the majority of the cases and occasionally intraoperatively according to intraoperative findings during and after the fashioning of the anastomosis. Loop ileostomy was anchored to the skin by suturing the edge of the proximal and distal ileal lumen to the skin.

Prior to ileostomy closure, the integrity of colo-rectal or colo-anal anastomosis was assessed by rectoscopy and gastrografin enema. Ileostomy closure was most commonly performed via an incision around the stoma, and occasionally through a laparotomy, and this was at the individual surgeon’s preference. Ileostomy was closed either with sutures by hand or with the use of stapling devices. The skin at the site of ileostomy was closed either vertically or with purse-string sutures.

Through reviewing the medical records of patients with low rectal cancer with TME-LARR and loop ileostomy, data concerning demographics, clinical characteristics, technical operative details, overall morbidity, ileostomy fashioning-related complications, and complications after ileostomy closure were collected for analysis. The presence of abdominal bloating and colicky pain, and/or nausea and vomiting, anorexia and/or reduced discharge of ileal content for more than 3 days was defined as ileus following loop ileostomy. The application of an ileostomy bag and peri-ileostomy skin care was graded as uneventful, problematic (occasional problems with dermatitis and bag application) or poor (inability to control leak of ileal content).

We conducted this study in compliance with the principles of the Declaration of Helsinki. This study’s protocol was reviewed and approved by the Institutional Review Board of all four hospitals (Ethic Committee of Nicosia General Hospital; Approval Code: NGHRC76/22 Approval Date: 19 December 2022). The requirement for informed consent was waived due to the retrospective nature of the study.

Statistical analysis was performed using the SPSS 27.0 (IBM Corp. Released 2016. IBM SPSS Statistics for Windows, Version 27.0. Armonk, NY, USA: IBM Corp.) statistical package. After checking the data for integrity, duplicate records and outliers, descriptive statistics were obtained, and graphs were plotted (bar charts, pie charts, box plots). Categorical variables were analyzed with Pearson’s Chi-Square, with Yates correction for cells with an expected frequency of less than five. Continuous variables were tested for normality using the Shapiro–Wilk test and then compared with Student’s *t*-test for independent samples, or Welch’s *t*-test in the case of unequal variances. Multivariate analysis was carried out using binary logistic regression. Odds ratios and 95% confidence intervals for each covariate were calculated. The level of statistical significance was set at 5% (*p* = 0.05).

## 3. Results

**Figure 1:**
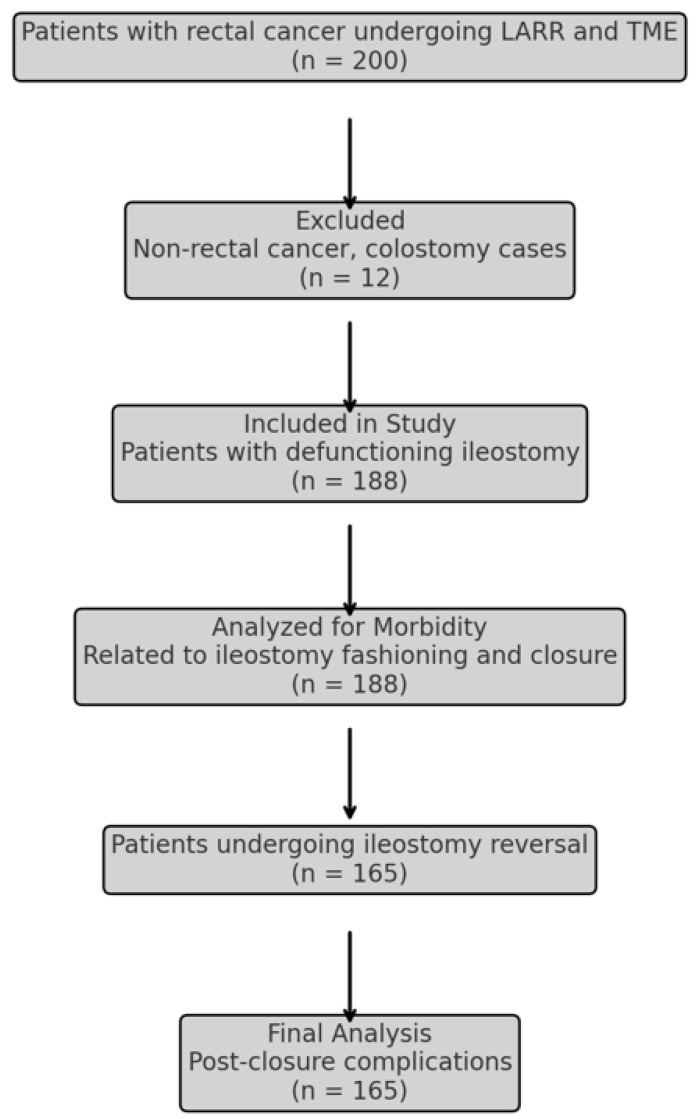
Flowchart of the selection of patients’ records.

After searching all the records of rectal cancer patients operated in the aforementioned centres, there were 188 consecutive patients with rectal cancer, subjected to TME-LARR and defunctioning ileostomy, identified from the records (Figure 1). The demographics, clinical characteristics and preoperative and operative data are shown in Table 1. Male patients comprised approximately 63% of the study population, whilst more than 1/5 of the sample was obese. Almost 46% of them had a long course of chemoradiotherapy. The median follow up was 38 (range: 6–66) months. LARR-TME was attempted with the laparoscopically assisted approach in 36% of the patients with a 12% rate of conversion to open. A stapled colo-rectal or colo-anal anastomosis was fashioned in most of the patients, whilst a hand-sewn anastomosis was performed in 7% of the cases who had an intersphincteric resection of the rectum. The median level of anastomosis was at 3.5 cm from the anal verge.

Three patients died immediately postoperatively: one from a cardiac ischemic attack, one from severe respiratory insufficiency, and the third from acute renal failure because of severe dehydration. Overall morbidity was 47%. Thirty-five patients (19%) developed an anastomotic leak, subclinically manifested and identified at rectoscopy or gastrografin enema.

Morbidity related to ileostomy fashioning was as high as 46%, and as mentioned above, one patient died because of dehydration and acute renal failure. Ileus, in the form described above, was seen in 38% of the cases, lasted for five days, at a median value (range: 3–14 days), and was treated conservatively in all but three cases (96%). Ileus occurred in only nine cases (5%) after the removal of the bridge at 12 to 14 days after fashioning. Other postoperative complications included dehydration in 19% of the cases, wound infection in 17%, chest pathology in 13%, urinary retention in 10% and wound hernia in 7% (Table 2). Dehydration was primarily manifested within two to three weeks after ileostomy fashioning. Only three cases presented with dehydration at the fourth week postoperatively. In total, 15% of the patients were readmitted because of various reasons, most common of which were dehydration (55%) and/or ileus (89%). The most common long-term complication from ileostomy, in the interval until closure, was the herniation of small bowel. Stoma care was problematic or poor in 23% of the cases, requiring several visits to stoma therapists (Table 3).

Through multivariate analysis, male gender and BMI were statistically significantly associated with increased ileostomy-related morbidity. Other parameters, such as neo-adjuvant treatment or age were not related to increased ileostomy morbidity (Table 4a). As far as post-closure ileostomy morbidity is concerned, the only factor affecting the morbidity based on multivariate analysis was the way of the skin closure of the ileostomy site (Table 4b) as it affected the ileostomy closure wound infection rate significantly with vertical closing having a 20 times higher chance of a wound infection (Odds ratio: 20.632; 95% CI: 1.614–263.744; *p* = 0.02).

Twenty-three patients (12.2%) failed to have their ileostomy reversed. The main reasons for failure were a persistent leak of primary anastomosis (9/23–39%) out of which one had a local recurrence, patients’ refusal (6/23–26%), disseminated metastatic disease (4/23–17.4%) and death (3/23–13%) and one patient was unfit to undergo reversal (1/23–4.4%). The median stoma period was 22 weeks (4–84). The main reasons for late reversal were adjuvant chemotherapy and anastomotic leak. In only six cases (3.6%), closure was performed through laparotomy. The median duration of surgery was 55 min (range: 20–125). Through univariate analyses, the duration of surgery was significantly related to the interval to closure (*p* < 0.01) and to BMI > 25 (*p* = 0.02). In 75% of the cases, anastomosis closure was performed with the use of stapling devices, or with sutures in the remaining patients. One patient died because of anastomotic leak. Overall closure morbidity was 16%. Eight patients (4.8%) presented with anastomotic leak, which was treated by reoperation in five (one mortality) and conservatively in the remaining three. Through univariate and multivariate analyses, no factor, such as anastomosis fashioning or adjuvant chemotherapy, was identified to predispose to anastomotic leak. Wound infection was most commonly seen in patients with vertical skin closure (*p* = 0.02). Wound hernia was seen in 9% of the cases attending post-reversal follow-up (range: 6–60 months) (Table 3).

## 4. Discussion

The results of this study corroborate the high morbidity associated with loop ileostomy formation following low anterior resection (LAR) with total mesorectal excision (TME) for rectal cancer, with an overall morbidity rate of 46%. Similar findings have been reported across recent studies, with morbidity rates ranging from 30% to 66% in larger cohorts [23,24,25,26,29,30]. This includes both early complications such as ileus and dehydration, as well as late-stage issues like herniation and prolapse, all of which can significantly impact patient outcomes [27,28,31,32].

### 4.1. Ileus and Dehydration

Postoperative ileus, affecting over one-third of the patients, remains a major concern in ileostomy management, especially as it predisposes patients to dehydration and subsequent renal complications. Our findings align with other studies, which identify male sex and obesity as independent risk factors for postoperative ileus [30,33,34,35,36]. Obesity, in particular, is a well-documented risk factor for ileus, possibly due to the increased abdominal pressure exerted on the mesenteric vasculature, leading to transient ischemia [30,37].

### 4.2. Risk Factors for Morbidity

Although age and neoadjuvant chemoradiotherapy are often implicated as risk factors for increased postoperative morbidity, our multivariate analysis did not identify these as significant in our cohort. Recent evidence suggests that the impact of these factors may be mitigated by advances in perioperative care, including enhanced recovery after surgery (ERAS) protocols, which have been shown to reduce morbidity related to fluid imbalance and ileus [29,33,38]. Nevertheless, male gender and obesity were confirmed as major contributors to ileostomy-related morbidity, as also observed in other contemporary studies [39,40,41].

### 4.3. Ileostomy Closure

The morbidity associated with ileostomy reversal, including wound infections and anastomotic leaks, was found to be 16% in our study, lower than in some previous reports that suggest rates of 20% to 30% [25,42,43,44]. The technique of skin closure played a significant role, with vertical closure being associated with a higher risk of infection compared to purse-string closure. This observation has been consistently reported in the literature, with recent studies advocating for purse-string closure to reduce the incidence of wound infection and promote faster healing [45,46,47].

### 4.4. Timing of Reversal

The median duration to ileostomy reversal in our cohort was 22 weeks, primarily influenced by adjuvant chemotherapy and anastomotic healing. Current guidelines suggest that early reversal, within 2 to 4 weeks in select patients with an intact anastomosis, may minimize ileostomy-related morbidity and improve long-term outcomes [34,38,48,49,50]. Furthermore, early reversal can prevent complications such as dehydration and malnutrition, which are particularly problematic in elderly or frail patients [38,51].

### 4.5. Limitations

While this study sheds light on the complications associated with defunctioning ileostomy after rectal cancer surgery, it is important to acknowledge some limitations. Since this study was retrospective, it relied heavily on the accuracy of medical records, which could introduce some bias. Additionally, the patients were treated across multiple centres, which means that there may have been variations in how surgeries were performed and how care was provided after the procedure. This makes it a bit harder to apply the results universally. The sample size, though respectable, may not have been large enough to detect more subtle differences in outcomes, especially in specific groups like older patients or those who underwent laparoscopic surgery. Additionally, while parameters such as blood chemistry and complete blood count (CBC) profiles could provide deeper insights into patient outcomes, these were not included in the analysis due to incomplete data across the cohort. Future studies will aim to address this gap by ensuring comprehensive data collection for these critical variables.

Another limitation of this study is that we did not analyze the timing of ileostomy closure using a time-to-event framework, such as Cox proportional hazards models. This type of analysis could better identify factors influencing closure timing, but due to the retrospective nature of the study, the time-point data necessary for such an analysis were not uniformly available. This approach will be considered in future prospective studies to explore these variables more thoroughly.

Another point to consider is that we did not evaluate patient-reported outcomes, such as quality of life or long-term effects, which would have given us a better idea of how these complications really impact daily living. Finally, while we analyzed several factors contributing to complications, other factors—like nutritional status or existing health conditions—may not have been fully explored.

## 5. Conclusions

The high morbidity associated with defunctioning ileostomy, particularly in male and obese patients, underscores the importance of careful patient selection and postoperative management. While the protective role of ileostomy in preventing anastomotic leaks remains undisputed, efforts should be made to mitigate its complications, including dehydration and wound infection. The early reversal of ileostomy, where feasible, represents a promising strategy for reducing the long-term complications associated with this procedure

## Figures and Tables

**Table 1:** Demographics, clinical characteristics and preoperative and operative data of the included patients.

No. of Patients Included	188
Gender: No. Male/Female; (%)	118/62; (62.8/37.2)
Age (Years)	66 (30–86)
BMI (Kg/m^2^) (167 observations) No. (%) of Pts: BMI > 30	28 (19–45) 37 (22.2%)
Distance of Tumour Lower Edge From Anal Verge (cm)	7 (2–10)
Preoperative T Stage [No. (%)]	T0,1: 13 (6.9); T2: 54 (28.7) T3: 106 (56.4); T4: 15 (8)
Preoperative N Stage [No. (%)]	N0: 126 (67); N1: 48 (25.5); N2: 14 (7.5)
Preoperative M Stage No. (%)	M0: 181 (96.3); M1: 7 (3.7)
Locally Advanced No. (%)	127/188 (67.6)
Tumour Differentiation	Good: 145 (77.1); Poor: 43 (22.9)
Neo-Adjuvant CRT [No. (%)]	Yes: 103/188 (54.8)
Surgical Approach [No. (%)]	Open: 120 (63.8); Lap: 68 (36.2)
Lap Converted to Open [No. (%)]	8/68 (11.8)
Fashioning of Anastomosis [No. (%)]	Stapled: 175 (93.1); Hand-Sawn: 13 (6.9)
Level of Anastomosis Distance from Anal Verge (cm)	3.5 (0.5–7)

BMI: body mass index; CRT: chemoradiotherapy; Lap: laparoscopic.

**Table 2:** Postoperative outcomes and treatments.

Mortality (3-month) (%)	3/188 (1.6)
Morbidity (%)	89/188 (47.3)
Anastomotic Leak (%)	35/188 (18.6)
Bleeding (%)	3/188 (1.6)
Obstruction (%)	2 (1.1)
Wound Infection (%)	32/188 (17)
Open Approach (%)	27/120 (23)
Lap Approach (%)	5/68 (7.4)
Wound Dehiscence (%)	2/188 (1.6)
Open Approach (%)	2/120 (1.7)
Lap Approach (%)	0/68 (0)
Wound Hernia (%)	14/188 (7)
Open Approach (%)	11/120 (11.2)
Lap Approach (%)	3/68 (4.4)
Cardiac Complications (%)	4/188 (2.1)
Open Approach (%)	4/120 (3.3)
Lap Approach (%)	0/68 (0)
Respiratory Complications (%)	24/188 (12.8)
Open Approach (%)	20/120 (16.7)
Lap Approach (%)	4/68 (5.9)
Urinary Retention (%)	18/188 (9.6)
Open Approach (%)	8/120 (6.7)
Lap Approach (%)	10/68 (14.7)
Adjuvant Radiotherapy (%)	4/185 (2.2)
Adjuvant Chemotherapy (%)	85/185 (46.5)

**Table 3:** Ileostomy-related complications.

**Ileostomy Fashioning**	
Mortality (%)	1/186 (0.5)
Morbidity (%)	100/186 (46.2)
Ileus (%)	71/186 (37.4)
Ileus Duration (d)	5 (2–15)
Ileus Treatment	
Conservative (%)	68/71 (95.8)
Surgical (%)	3/71 (4.2)
Dehydration (%)	35/186 (18.8)
Acute Renal Failure (%)	7/186 (3.8) [7/35 (20)]
Readmission (%)	27/186 (15.4)
Herniation (%)	25 (13.5)
Prolapse (%)	10/185 (5.4)
Inversion (%)	3/185 (1.6)
Stoma Care	
Uneventful (%)	143/185 (77.3)
Problematic (%)	29/185 (15.7)
Poor (%)	13/185 (7)
**Ileostomy Reversal**	
Yes/No (%)	165/23 (87.8/12.2)
Interval to Closure (w)	22 (4–84)
Mortality (%)	1/165 (0.6)
Morbidity (%)	27/165 (16.4%)
Closure Fashioning	
Stapled (%)	123/165 (74.5)
Hand-Sawn (%)	42/165 (25.5)
Duration of Surgery (min)	55 (30–125) (153 pts)
Skin Closure	(153 pts)
Vertical (%)	116/165 (69.9)
Purse-String (%)	49/165 (30.1)
Wound Length (cm)	(153 pts)
Vertical	5 (2–8)
Purse-String	1 (0.5–1.7)
Ileus (%)	17/165 (10.3)
Anastomotic Leak (%)	8/165 (4.8)
Wound Infection (%)	21/165 (12.7)
Vertical (%)	20/116 (17.2)
Purse-String (%)	1/49 (2)
Hospital Stay (d)	3 (2–27)
Wound Hernia (%)	13/144 (9) (144 pts)

**Table 4:** (**a**). Multivariate analysis of factors affecting stoma-related morbidity. (**b**). Multivariate analysis of factors affecting stoma closure-related morbidity.

(**a**)		
**Factor**	**Odds Ratio (95% CI)**	***p*-Value**
Sex	Female 1	
	Male 2.707 (1.396, 5.249)	0.003 *
Age (years)	1.005 (0.976, 1.034)	0.747
BMI (kg/m^2^)	1.093 (1.012, 1.180)	0.023 *
Neoadjuvant treatment	No 1	
	Yes 1.484 (0.755, 2.917)	0.252
(**b**)		
**Factor**	**Odds Ratio (95% CI)**	***p*-Value**
Sex	Female 1	
	Male 2.466 (0.912, 6.667)	0.075
Age (years)	0.978 (0.939, 1.019)	0.287
BMI (kg/m^2^)	0.955 (0.853, 1.069)	0.425
Neoadjuvant treatment	No 1	
	Yes 1.308 (0.488, 3.509)	0.593
Ileostomy site skin wound closure	Purstring 1	
	Vertical 15.535 (2.02, 119.466)	0.008 *
Ileostomy site wound length (cm)	0.862 (0.621, 1.197)	0.375
Closure (weeks)	1.022 (0.989, 1.057)	0.177
Adjuvant radiotherapy	No 1	
	Yes 0.171 (0.007, 4.206)	0.28
Adjuvant chemotherapy	No 1	
	Yes 0.718 (0.211, 2.448)	0.597

* Statistically significant variables.

## Data Availability

Data can become available on request provided the approval of the IRB is obtained as the IRB did not allow for the data to be publicly available.
